# Ultrasound‐mediated delivery of flexibility‐tunable polymer drug conjugates for treating glioblastoma

**DOI:** 10.1002/btm2.10408

**Published:** 2022-11-19

**Authors:** Tao Sun, Vinu Krishnan, Daniel C. Pan, Sergey K. Filippov, Sagi Ravid, Apoorva Sarode, Jayoung Kim, Yongzhi Zhang, Chanikarn Power, Sezin Aday, Junling Guo, Jeffrey M. Karp, Nathan J. McDannold, Samir S. Mitragotri

**Affiliations:** ^1^ John A. Paulson School of Engineering and Applied Sciences Harvard University Cambridge Massachusetts USA; ^2^ Wyss Institute for Biologically Inspired Engineering, Harvard University Boston Massachusetts USA; ^3^ Focused Ultrasound Laboratory, Department of Radiology Brigham and Women's Hospital, Harvard Medical School Boston Massachusetts USA; ^4^ Department of Anesthesiology Perioperative and Pain Medicine, Brigham and Women's Hospital, Harvard Medical School Boston Massachusetts USA; ^5^ Center for Nanomedicine, Harvard Stem Cell Institute, Brigham and Women's Hospital, Harvard Medical School Boston Massachusetts USA; ^6^ Harvard‐MIT Division of Health Sciences and Technology Cambridge Massachusetts USA; ^7^ Proteomics Platform, Broad Institute of MIT and Harvard Cambridge Massachusetts USA; ^8^ Present address: Pharmaceutical Sciences Laboratory Åbo Akademi University, Turku Bioscience Turku Finland; ^9^ Present address: College of Biomass Science and Engineering Sichuan University Chengdu Sichuan China

**Keywords:** chemotherapy, drug delivery, focused ultrasound, glioblastoma, hyaluronic acid

## Abstract

Effective chemotherapy delivery for glioblastoma multiforme (GBM) is limited by drug transport across the blood–brain barrier and poor efficacy of single agents. Polymer–drug conjugates can be used to deliver drug combinations with a ratiometric dosing. However, the behaviors and effectiveness of this system have never been well investigated in GBM models. Here, we report flexible conjugates of hyaluronic acid (HA) with camptothecin (CPT) and doxorubicin (DOX) delivered into the brain using focused ultrasound (FUS). In vitro toxicity assays reveal that DOX‐CPT exhibited synergistic action against GBM in a ratio‐dependent manner when delivered as HA conjugates. FUS is employed to improve penetration of DOX‐HA‐CPT conjugates into the brain in vivo in a murine GBM model. Small‐angle x‐ray scattering characterizations of the conjugates show that the DOX:CPT ratio affects the polymer chain flexibility. Conjugates with the highest flexibility yield the highest efficacy in treating mouse GBM in vivo. Our results demonstrate the association of FUS‐enhanced delivery of combination chemotherapy and the drug‐ratio‐dependent flexibility of the HA conjugates. Drug ratio in the polymer nanocomplex may thus be employed as a key factor to modulate FUS drug delivery efficiency via controlling the polymer flexibility. Our characterizations also highlight the significance of understanding the flexibility of drug carriers in ultrasound‐mediated drug delivery systems.

**Abbreviations**
BBB: blood–brain barrier
BTB: blood‐tumor barrier
CPT: camptothecin
DOX: doxorubicin
FUS: focused ultrasound
GBM: glioblastoma multiforme
H&E: hematoxylin and eosin
HA: hyaluronic acid
IHC: immunohistochemistry
MRI: magnetic resonance imaging
SAXS: small‐angle x‐ray scattering

## 1 INTRODUCTION

Glioma is the most common primary tumor of the central nervous system. Nearly half of glioma patients develop the most aggressive form of the disease, glioblastoma multiforme (GBM; grade IV glioma). GBM carries a notoriously poor prognosis and adult diffuse GBM is resistant to all currently therapies.^1^

Among various experimental therapies, a topoisomerase (Top) II inhibitor, doxorubicin (DOX) or its liposomal form (Doxil), has been extensively investigated for the treatment of GBM.^2^, ^3^, ^4^, ^5^, ^6^, ^7^, ^8^, ^9^ Since the entry of intravenously injected therapies into the brain is limited by the blood–brain barrier (BBB) and the blood‐tumor barrier (BTB),^10^, ^11^ focused ultrasound (FUS), a clinically viable tool, has been tested to transiently open the BBB and BTB.^11^ While several preclinical reports demonstrate the efficacy of FUS‐enhanced DOX delivery, the results have been variable,^9^ and this strategy has not been used in humans except for one single study.^12^ Another topoisomerase (I) inhibitor, irinotecan (Camptothecin‐11 [CPT‐11]) has also been successfully delivered to a rat GBM model after FUS‐enabled BBB opening. However, the treatment did not achieve sufficient efficacy to improve the survival of F98 glioma model.^13^

Similar concerns of the lack of efficacy in single agent chemotherapies have been raised in treating various other types of cancer. To address these issues, we^14^ and others^15^ have demonstrated that a combination of two Top inhibitors, DOX and CPT, exhibits a highly synergistic activity against various cancers including brain tumors.^16^, ^17^ CPT and DOX can inhibit both Top I and II enzymes that regulate DNA transcription and cell replication, and this combination strategy could potentially offer a higher efficacy than a single agent therapy. Studies have indicated increased levels of Top I enzymes in glioma cells in contrast to the normal brain tissue,^18^, ^19^, ^20^, ^21^, ^22^ suggesting a selective activity of CPT in brain cancer.^18^ Previous works also suggest that the DOX‐CPT ratios in the drug combination would affect the treatment efficacy.^14^, ^23^ However, success in translating the combination of DOX‐CPT into the clinic has been hindered primarily due to solubility issues associated with CPT and the ability to deliver synergistic DOX‐CPT ratios to the target site. We address both issues by conjugating CPT and DOX to HA, thereby improving the solubility of CPT and preserving the drug ratios. In addition, FUS enables spatially targeted delivery of these precise drug ratios to the tumor, ensuring synergistic antitumor activity of CPT and DOX at the site of action.

As we use FUS for physically enhancing drug transport across the BBB, it is also vital to understand the association between the delivery efficiency and the physical characteristics of the HA conjugates, which can be affected by the DOX‐CPT drug ratio. While previous reports using FUS for drug delivery to the brain only demonstrated that the size of the drug/agent could affect the delivery under the same ultrasound parameters,^24^, ^25^ other physical parameters of the drug carrier can also play an important role and have not been explored. Additionally, previous works did not study this relationship using polymer conjugates. We address these issues by using small‐angle x‐ray scattering (SAXS) characterization of the conjugates to decipher the relationships among DOX:CPT ratio, the polymer chain flexibility, and the drug delivery efficiency, and efficacy showed in the animal studies.

Here, we evaluated HA conjugates of DOX and CPT for the treatment of GBM. To co‐deliver these two drugs at specific ratios, we used HA as the drug carrier^14^ for its biocompatibility and the targeting specificity for CD44, which is over‐expressed on human GBM cells.^26^ Based on an in vitro cell toxicity assay, in vivo drug delivery, and treatment efficacy evaluation in a murine GBM model GL261, we identified a synergistic combination of Top I (CPT) and Top II inhibitors (DOX) at specific ratios in the HA conjugates. Leveraging previous work demonstrating the ability of FUS to enhance chemotherapeutic delivery into the GBM,^3^, ^4^, ^5^, ^6^, ^7^, ^8^, ^27^ we demonstrate that FUS enables the delivery of HA‐CPT‐DOX conjugates into the brain for the treatment of GBM. We further show that SAXS characterizations of the conjugates reveal that DOX:CPT ratio affects the polymer chain flexibility. Conjugates with the highest flexibility (lowest stiffness) yielded the highest efficacy in treating mouse glioblastoma in vivo after FUS BBB opening.

## 2 RESULTS

### 2.1 Synthesis and characterization of hyaluronic acid conjugates

**FIGURE 1:**
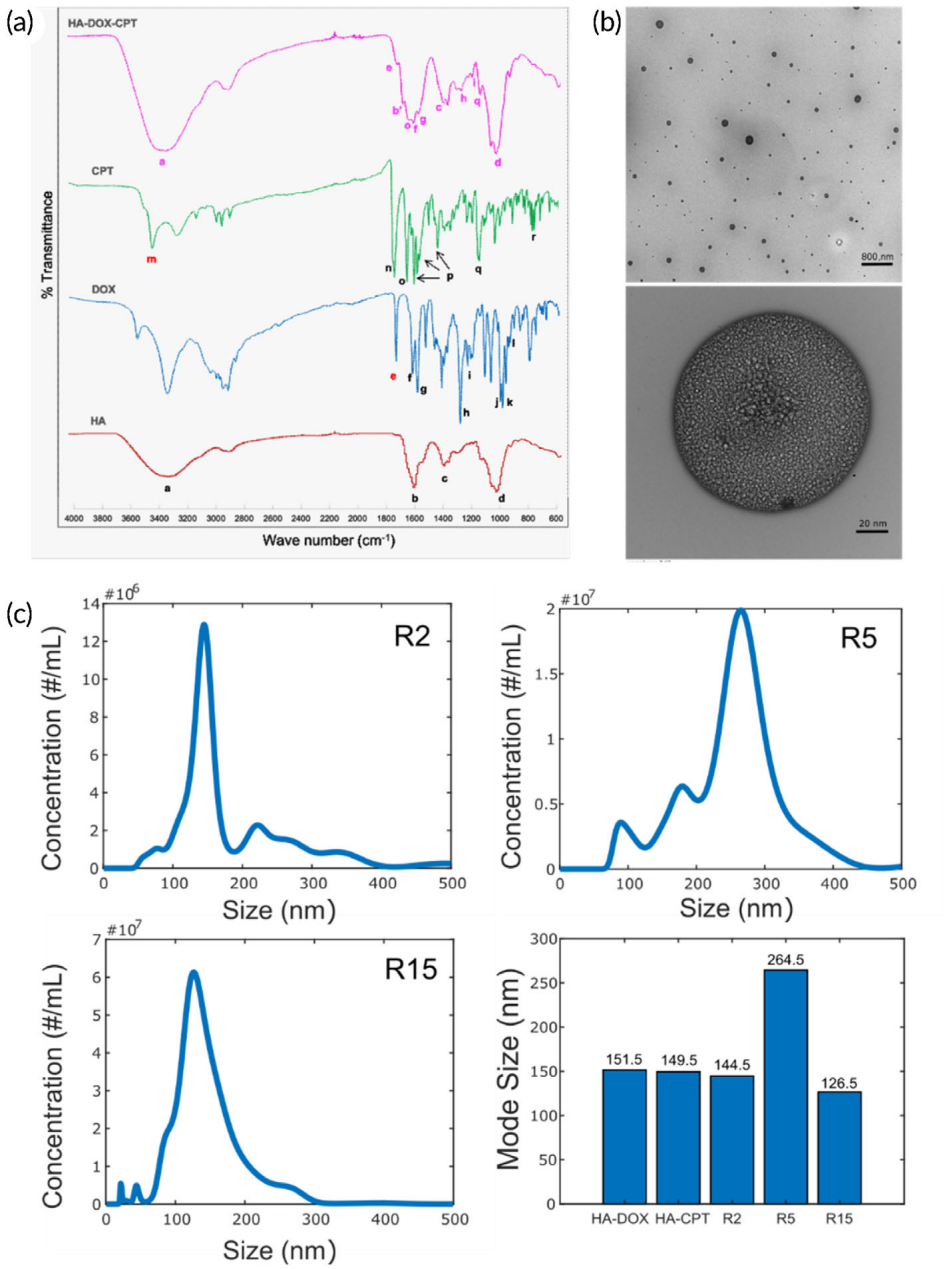
Physical characterization of DOX‐HA‐CPT. (a) A relative comparison of the FTIR spectra of native HA, free DOX, free CPT and DOX‐HA‐CPT confirmed the covalent drug conjugation to the HA polymer. (b) TEM imaging of DOX‐HA‐CPT (R15) revealed a particle‐based structure. (c) Size distribution characterization in nanoparticle tracking analyses of DOX‐HA‐CPT conjugates

DOX and CPT were chemically conjugated to HA forming single drug conjugates or the dual drug conjugates at 3 molar ratios (HA‐DOX‐CPT at R2, R5 and R15, R = molar ratio of DOX:CPT in the conjugate) (Table S1). The amount of each drug conjugated to HA was measured using the fluorescence spectra that was specific for each molecule (Table S2). Fourier transform infrared spectroscopy (FTIR) confirmed the formation of amide and ester bonds suggesting the successful covalent conjugation of CPT and DOX to HA (Figure 1a). The 50‐kDa HA was conjugated to the hydrophobic drugs CPT and DOX. The covalent linkage to HA was validated using FTIR, as shown in Figure 1. For HA, characteristic peaks are observed due to the stretching vibration of —OH and —NH groups (3309 cm^−1^) (a), and the symmetric and asymmetric vibration of COO— (1613 cm^−1^) (b) and 1400 cm^−1^ (c), respectively. Furthermore, the characteristic peak at 1030 cm^−1^ (d) is attributed to the C—O—C hemiacetalic saccharide linkages in the polymeric chain.^28^ Characteristic peaks for pure DOX were observed owing to the stretching vibrations of primary amines at 1730 cm^−1^ (e), ring stretching and CO—H bonds at 1616.08 cm^−1^ (f), Vibrations of aromatic C—H bonds at 1580 cm^−1^ (g), O—H bending, ring, C—OH, C—H bonds at 1284.4 cm^−1^ (h) and 1210 cm^−1^ (i), aliphatic C—H, C—OH, and C—C=O at 985 cm^−1^ (j), C—H bending, CO—C and O—C—O bonds at 960 cm^−1^ (k), and CO—H and C—H bending at 916 cm^−1^ (l).^29^ FTIR spectrum of free CPT shows absorption bands at 3423.1 cm^−1^ (m), 1738.1 cm^−1^ (n), 1648 cm^−1^ (o), 1597, 1578.5, 1436.5 cm^−1^ (p) and 1150 cm^−1^ (q) corresponding to the hydroxyl (—OH), C—O stretch for cyclic ester (lactone), carbonyl (C=O) stretching for pyridine, C=C, C=N stretching for quinoline ring and C—C(=O)—O stretching, respectively. The peak at 767 cm^−1^ (r) represents contribution from the adjacent hetero‐aromatic nuclei.^30^

The FTIR spectrum for HA‐DOX‐CPT suggested that the polysaccharide structure of HA backbone was intact. While the spectrum also confirms the presence of DOX through its strong characteristic peaks (f, g, h, i), the disappearance of the peak for primary amines confirmed the reaction with COOH groups of HA. Similarly, the characteristic peaks “o” and “q” from CPT remain unaffected in the FTIR spectrum of HA‐DOX‐CPT. Peak “m” corresponding to hydroxyl groups disappears indicating their reaction with HA to form ester linkages. Formation of ester bond results in shift of the peak for carbonyl from 1613 cm^−1^ (b) to 1686 cm^−1^ (b′).

Morphology and size of the conjugates were examined via transmission electron microscopy (TEM) **(**Figure 1b), atomic force microscopy (AFM) (Figure S1 and Table S3) and nanoparticle tracking analyses (NTA) (Figure 1c). TEM revealed a micellar appearance, suggesting that drug conjugation imparts hydrophobicity to HA resulting in the self‐assembly. NTA revealed the average mean size to be 151.5 nm for HA‐DOX; 149.5 nm for HA‐CPT; 144.5, 264.5, and 126.5 nm for R2, R5, and R15, respectively (Figures 1c and S2).

### 2.2 Cell viability study

**FIGURE 2:**
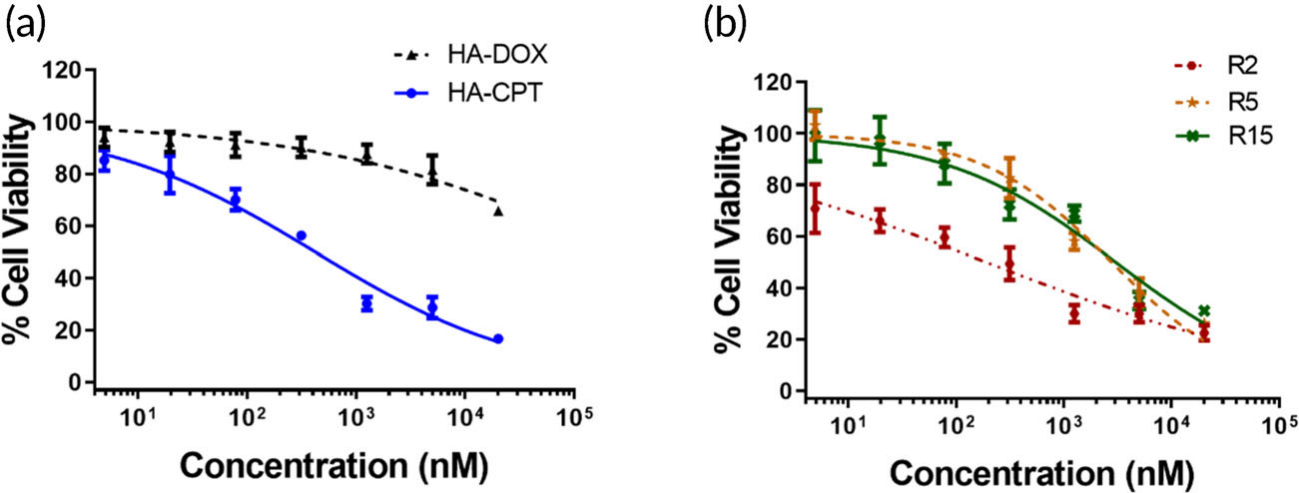
Cell viability in response to HA‐drug conjugates, including (a) HA‐CPT, HA‐DOX, and (b) R2, R5, and R15. R represents the molar ratio of DOX and CPT in the DOX‐HA‐CPT. Data correspond to mean ± SEM of three separate experiments.

To investigate the antitumor effects and optimize the drug ratio in the HA‐drug conjugates (DOX‐HA‐CPT), we performed a cell viability study. Cell viability results (Figure 2) demonstrate the combinatorial or synergistic effects of CPT and DOX in suppressing the growth of murine GL261 GBM cells, and the DOX:CPT ratio dependence on the efficacy. The half‐maximal inhibitory concentration (IC50) was used as a measure of the potency of DOX‐HA‐CPT in inhibiting GL261 cell growth. Combinational index (C.I.) values were determined using the Chou–Talalay method.

Compared to single drug conjugates, DOX‐HA‐CPT was highly effective against GL261 cells. When compared with the IC50 value for DOX in HA‐DOX, all three conjugates (R2, R5, and R15) were more potent compared to the single drug conjugate. Further, the IC50 values for CPT in R2 and R15 were approximately 4.4‐ and 2.1‐fold less than the IC50 for CPT in HA‐CPT alone. However, the IC50 value for CPT in R5 increased by 32% compared to HA‐CPT alone (Table 1). On this account, R2 and R15 were the only conjugates advanced for in vivo studies. Additionally, C.I. was estimated based on the IC50 values of single and dual‐drug treatment. A value of C.I. < 1 indicates synergism; C.I. = 1 corresponds to an additive effect; and C.I. > 1 shows antagonism. The highest synergistic interaction was observed for R2 (C.I. = 0.23), followed by R15 (C.I. = 0.5). R5 depicted a highly antagonistic interaction (C.I. = 1.33).

**TABLE 1:** Potency and synergism assessments of HA‐drug conjugates

	IC50		
HA‐CPT	420.6 nM ± 0.09		
HA‐DOX	260 mM ± 0.35		
	IC50 (CPT)	IC50 (DOX)	C.I.
R2	95.05 nM ± 0.10	190.1 nM ± 0.10	0.23 (synergistic)
R5	554 nM ± 0.17	2.8 μM ± 0.17	1.33 (antagonistic)
R15	202 nM ± 0.10	3 μM ± 0.10	0.5 (synergistic)

Abbreviations: CPT, camptothecin; C.I., combinational index; DOX, doxorubicin; IC50, half‐maximal inhibitory concentration.

### 2.3 FUS‐triggered drug delivery

**FIGURE 3:**
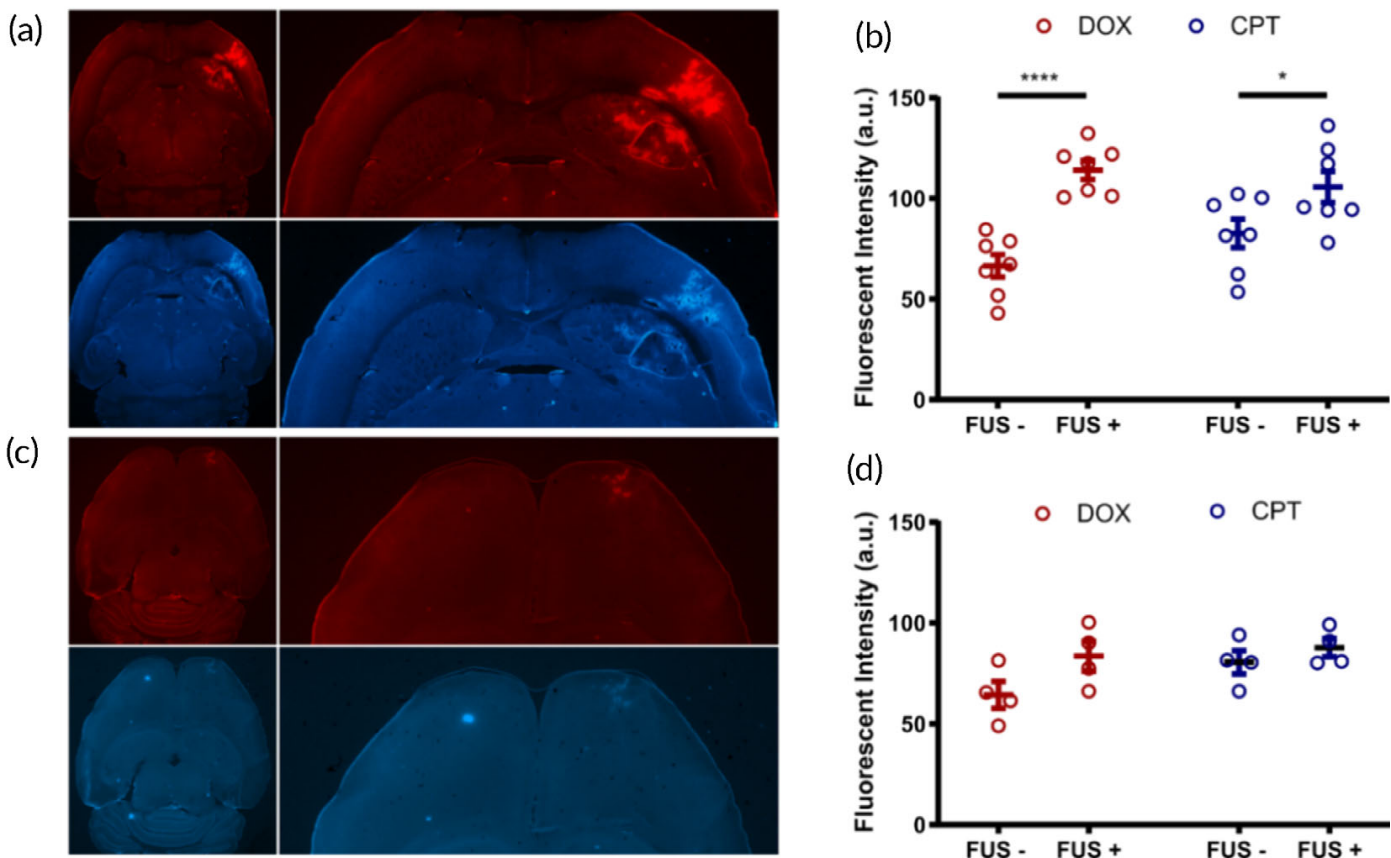
FUS‐triggered delivery of DOX and CPT via HA‐drug conjugates R15 (a, b, *n* = 7) and R2 (c, d, *n* = 4). FUS was applied on the striatum area of the right hemispheres. The contralateral sides were serving as the control (FUS‐groups). Data correspond to mean ± SEM. *p < 0.05, ****p < 0.0001

We next tested whether the candidate DOX‐HA‐CPT (R2 and R15), which showed synergistic antitumor effects in the cell viability study, can be delivered across the BBB in healthy mouse brains. Following one session of FUS‐enabled BBB opening together with DOX‐HA‐CPT intravenous administration (5 mg/kg of body weight, FUS started immediately after drug administration), wild‐type mice were sacrificed, and brain samples were harvested for fluorescence microscopic assessment. The R15 conjugates were delivered to the striatum areas **(**Figure 3a), even with limited plasma drug concentrations at the time of sacrificing the animals (<5% injected dose at 2 h post drug administration, see Figure S3). The delivery of both DOX and CPT was significantly enhanced post FUS treatment (Figure 3b). However, surprisingly, the enhancement of FUS‐triggered delivery of R2 was not as evident as that of R15 (Figure 3c). FUS‐treated areas exhibited moderately elevated delivery of R2, but the fluorescent intensity was not significantly higher compared to that of non‐FUS‐treated areas (Figure 3d). As R2 was delivered far less than R15, we cannot find evident hyperintense spots on the similar planes (which typically should be in the middle of the dorsal–ventral axis of the brain, as shown in Figure 3a) in the R2 group. The R2 delivery was mostly situated closer to the cortex regions due to a higher vascular density.

Of note, the two DOX‐HA‐CPT tested here contained the same amount of DOX, but R2 carries 7.5 times more CPT than R15. However, after FUS‐enabled BBB opening, R15 was able to deliver more of both chemotherapeutics compared to R2. Considering the same FUS settings were used in the two groups, we speculated that the DOX:CPT ratio may affect some properties of the DOX‐HA‐CPT relevant for FUS drug delivery efficiency. As shown in Figure 1c, R2 has a median size of 144.5 nm, while R15 shows a slightly smaller size distribution with a median size of 128.5 nm. Indeed, various previous reports suggest that FUS‐facilitated drug delivery across the BBB is much more efficient when the drug of interest carries smaller molecular weights. However, it is unlikely that the results found in Figure 3 can be solely explained by the slight size difference of the two DOX‐HA‐CPT candidates tested. It is possible that the DOX:CPT ratio controls other structural properties of DOX‐HA‐CPT that lead to such a drastic change in the delivery profiles after FUS‐enabled BBB opening.

### 2.4 Treatment efficacy

**FIGURE 4:**
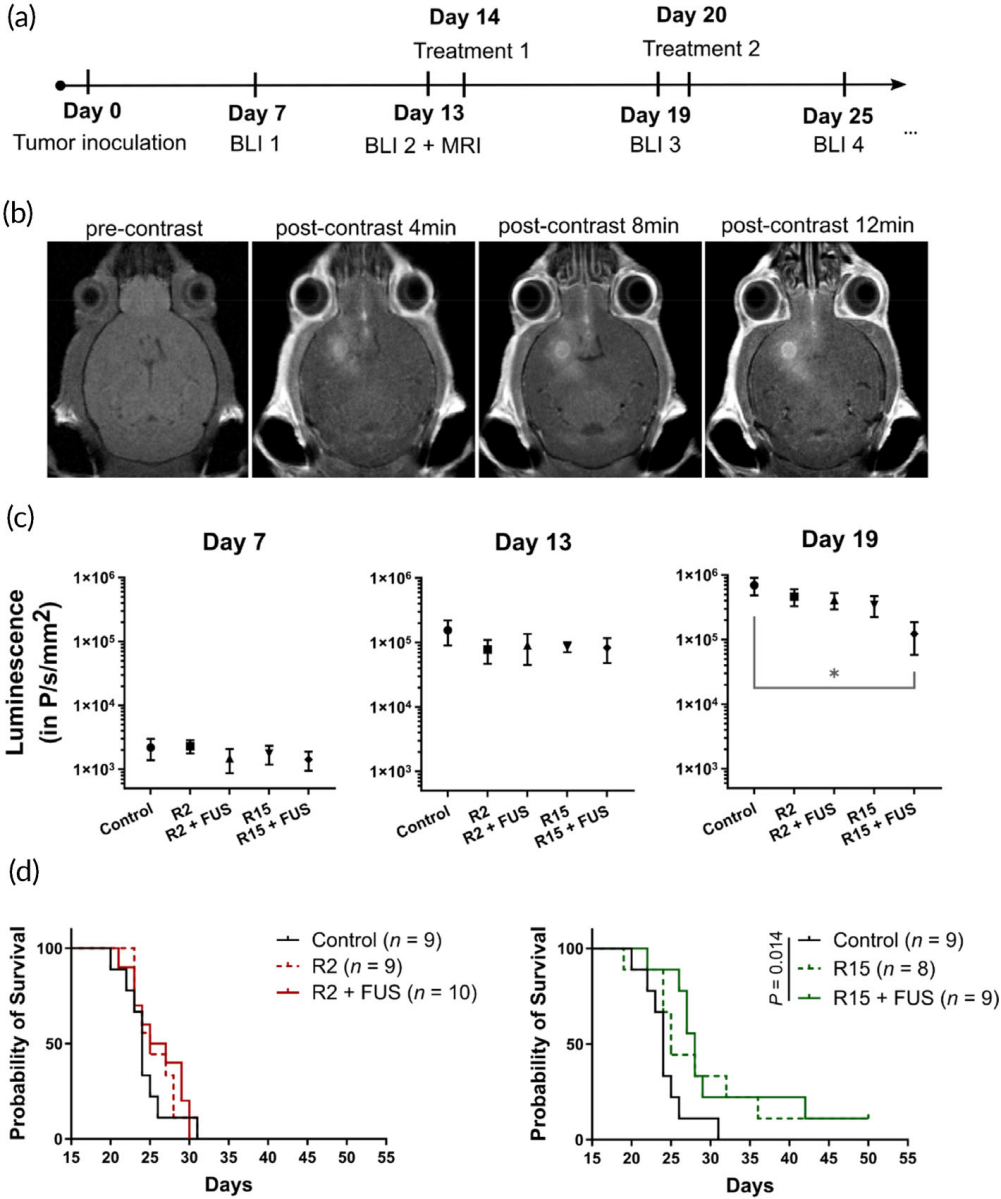
FUS‐enhanced R15 delivery inhibited tumor growth and improved survival. (a) Experimental protocol of the survival study. Treatment = nanoconjugates (R2 or R15) with or without FUS, BLI, bioluminescence imaging; MRI, magnetic resonance imaging. (b) T1‐weighted contrast MRI confirmed the BBB opening. The increased accumulation of MR contrast indicated the enhanced drug delivery after FUS treatment. (c) Bioluminescence imaging results showed that R15 + FUS combination therapy achieved significant tumor suppression 5 days after the FUS treatment (Day 19). (d) Survival curves of five experimental groups. Only R15 + FUS combination therapy significantly improved survival benefits compared to the control group. Data correspond to mean ± SEM. *p < 0.05

To test whether HA‐CPT‐DOX can be used for treating GBM, a survival study was performed in the GL261 model assessing the treatment efficacy of R2 and R15 with or without the FUS treatment (Figure 4a). After intracranial tumor inoculation, we monitored the tumor growth using bioluminescent imaging starting on Day 7. Two sessions of FUS treatment together with DOX‐HA‐CPT administration (5 mg/kg of body weight) were performed on Days 14 and 20. Magnetic resonance imaging (MRI) was also used on Day 13 to locate the tumor for FUS targeting and immediately after the first session of FUS to confirm the BBB opening (Figure 4b). We found that the R15 + FUS combination treatment was the only therapy that successfully inhibited tumor growth (Figure 4c) and improved the survival (Figure 4d).

Even though only FUS‐R15 combination therapy demonstrated significant tumor suppression and survival benefits in treating GL261 model, all treated groups exhibited improvement in several metrics compared to the control group (Table 1). FUS‐R15 treatment resulted in the longest median survival and the most number of mid‐term survivors among all the groups. Overall, R15 therapies (monotherapy or with FUS) were more efficacious than R2 therapies. This is in agreement with the conclusion of the cell viability assays based on the IC50 and C.I assessments (Table 2). Additionally, this in vivo efficacy result is consistent with the observation that more CPT and DOX was delivered via R15 than R2 after FUS‐enabled BBB opening (Figure 3).

**TABLE 2:** Treatment efficacy metrics in the survival study

	Control	R2	R2 + FUS	R15	R15 + FUS
Median survival (in Days)	24	25	26	26.5	28
Number of mid‐term survivors (Day 25)	3/9	5/9	6/10	6/8	8/9
Number of long‐term survivors (Day 50)	0/9	0/9	0/10	1/8	1/9

Abbreviation: FUS, focused ultrasound.

**FIGURE 5:**
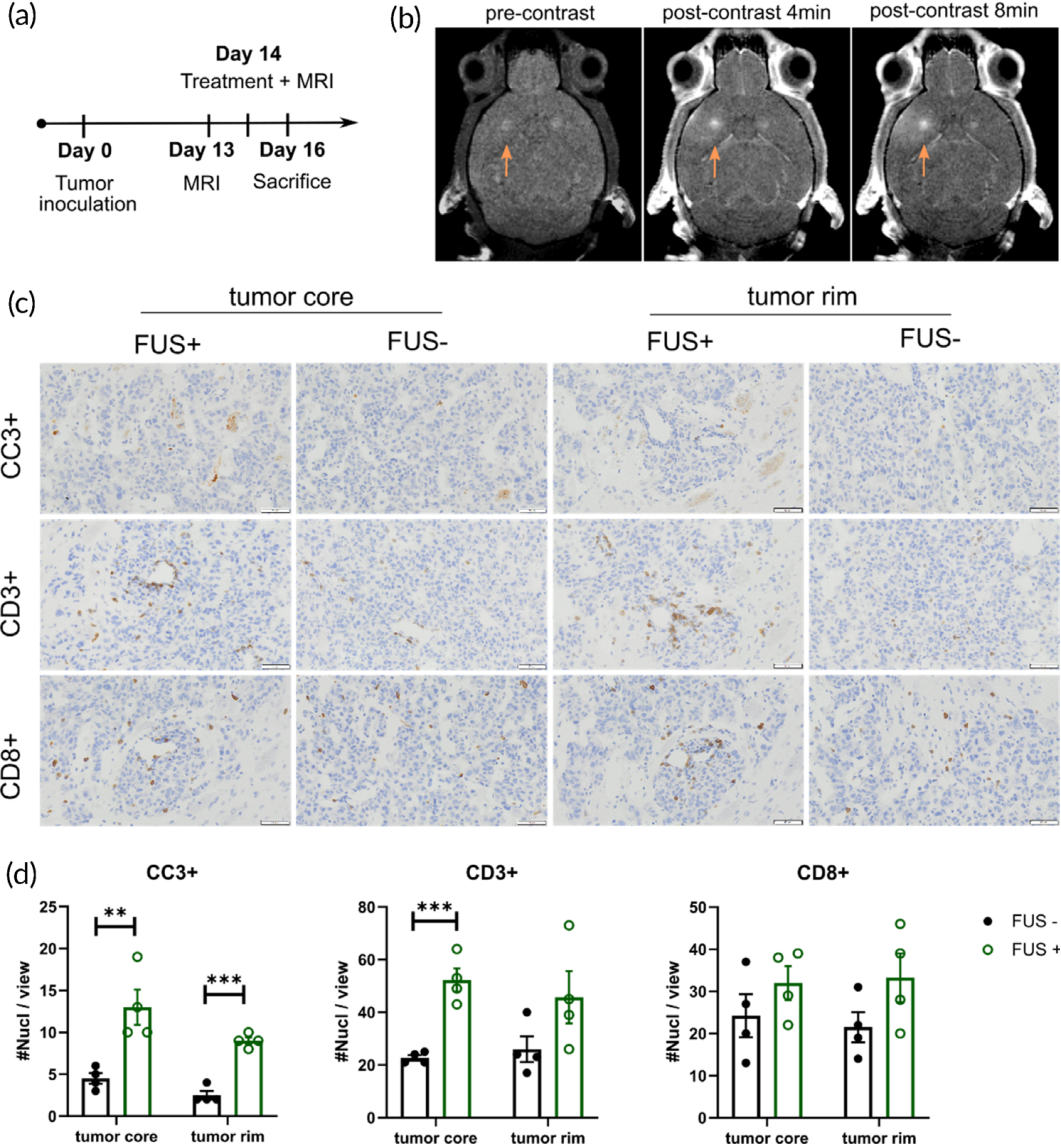
Histology study assessing apoptosis and T‐cell infiltration using a bilateral tumor model. All animals (*n* = 4) received systemic treatment of R15 with FUS applied on only one hemisphere. (a) Experimental protocol. (b) T1‐weighted contrast‐enhanced MRI confirmed that BBB opening enhanced the permeability of the sonicated tumor (arrowed). (c, d) Compared to R15 monotherapy, GL261 mice treated with FUS and R15 had increased apoptosis (CC3+) and enhanced T cell infiltration. Data correspond to mean ± SEM. **p < 0.01, ***p < 0.001

To further test whether FUS can enhance the delivery of chemotherapies locally to tumors, we performed immunohistochemistry (IHC) staining to quantify apoptosis in tumors. Tested in a bilateral tumor model under the protocol shown in Figure 5a, we only used R15 here as the DOX‐HA‐CPT to unveil the role of FUS in enhancing the treatment efficacy. T1‐weighted contrast MRI confirmed the BBB opening on the tumor with FUS application (arrows in Figure 5b) and enhanced drug delivery compared to the tumor on the contralateral side (FUS‐group, i.e., R15 only group). As shown in Figure 5c, R15 + FUS significantly increased the amount of apoptosis (cleaved caspase 3, CC3+) in both the tumor core and tumor rim, compared to R15 monotherapy. This result suggests that FUS led to a more effective release of chemotherapy into the parenchyma of GL261 tumors. In addition, we stained CD3 and CD8 in the brain sections to assess the T‐cell infiltration. We found that FUS significantly enhanced CD3+ T‐cell infiltration in the tumor core. In the tumor rim, the amount of CD3+ and CD8+ T cells was also elevated in response to FUS. However, this effect was not significant. This is not surprising as the tumor rim is more vascularized and may attract T cells without the additional permeabilization of FUS. In the tumor core, however, FUS showed enhanced T‐cell accumulation that may lead to an enhanced ability to eradicate tumor cells. Taken together, FUS‐R15 combination therapy was identified to enhance synergistic chemotherapy delivery and T‐cell infiltration, resulting in improved survival benefits in treating the GL261 GBM model.

### 2.5 Polymer flexibility

As described earlier, it was surprising to find R2, which was effective in the cell assay, had limited delivery after FUS‐triggered BBB opening compared to R15. To further address this question and link other physical properties of the polymers to the FUS‐trigged treatment outcomes, we used SAXS to characterize the polymer conformational properties.

**FIGURE 6:**
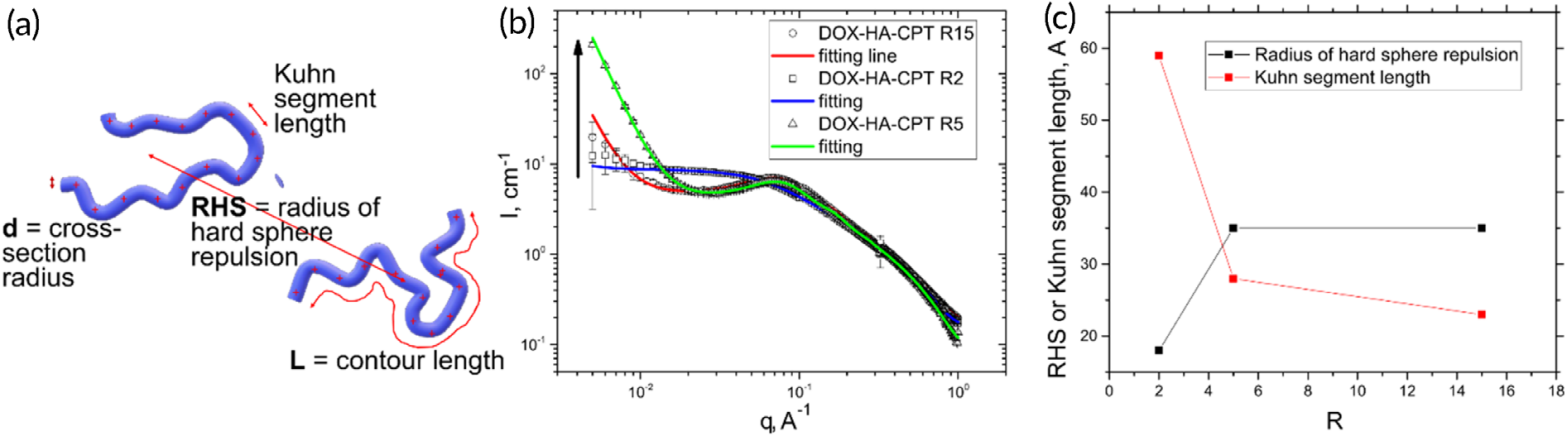
Small angle x‐ray scattering (SAXS) characterizations of DOX‐HA‐CPT in PBS. (a) The SAXS data were fitted to the model of Worm‐Like Chain to characterize the equilibrium rigidity of polymers (described by Kuhn segment length), and the interparticle interactions (described by radius of hard sphere repulsion, RHS). (b) Strong scattering intensities of the drug conjugates and their model fittings show the morphological transition on the polymeric chain in R5 and R15. *I* refers to the scattering intensity and *q* represents the scattering vector. (c) A clear relationship is identified between RHS and Kuhn segment length as a function of drug ratio, R.

SAXS was used to determine the equilibrium rigidity of DOX‐HA‐CPT in solution.^31^ The polymer equilibrium flexibility/rigidity can be described by the Kuhn segment length (Figure 6a).^32^ A strong scattering intensity observed for DOX‐HA‐CPT confirmed its particulate shape as seen with TEM (Figure 1b) and AFM (supporting materials S1). To evaluate the Kuhn segment length, the SAXS data were fit to a model of a “Worm‐Like Chain”^33^ describing the conformation of semi‐rigid and rigid macromolecules (Figure 6b). This model was chosen due to the charged nature of polymers in this study.

In addition, the form‐factors of aggregates and the hard sphere repulsion structure factor were applied to describe the interparticle interactions. Several structural parameters were extracted from the fitting including the values of Kuhn segment length and radius of hard sphere repulsion (RHS, Figure 6a).^32^ A clear correlation (Figure 6c) was observed between the RHS and Kuhn segment length as a function of drug ratio, R. Polymer conjugates with a higher ratio of DOX to CPT exhibit a lower Kuhn segment length and higher RHS. This suggests a more drastic morphological transition on polymeric chain with an increasing R. Therefore, in this study, the SAXS results demonstrated that R15 and R5 are flexible in nature, whereas R2 is a semi‐flexible polymer that is more rigid than R15 and R5.

## 3 DISCUSSION

In this study, CPT and DOX were delivered at specific ratios as DOX‐HA‐CPT conjugates after FUS‐enabled blood–brain/tumor barrier disruption for treating GBM in mice. We identified that DOX‐CPT synergy was associated with DOX:CPT ratio in the HA‐based nanoconjugates in vitro. In addition, another ratiometric dependency was also observed in FUS‐triggered local delivery of chemotherapeutics in vivo, leading to distinct treatment efficacy in a murine GBM model. However, conjugates that worked best in vitro were not most effective in vivo. The distinct behavior of conjugates in vivo and in vitro is particularly interesting, suggesting that the drug ratio in the polymer conjugates may affect some physical/structural parameter(s) that could potentially modulate the drug delivery outcomes under ultrasound triggering.

The most well‐accepted physical parameter that could directly affect the FUS‐enabled drug delivery across the BBB is the size of the particles.^24^, ^25^ TEM (Figure 1b), AFM (Figure S1 and Table S2) and NTA (Figure 1c) revealed a self‐assembled polydisperse spherical morphology for DOX‐HA‐CPT in PBS. However, based on the size distributions, both conjugates are within the same range (Figure 1c). SAXS results showed that the DOX:CPT molar ratio affects the HA polymer chain flexibility, and the most flexible version of DOX‐HA‐CPT exhibited the highest therapeutic efficacy in vivo. Additionally, based on the literature data, there exists evidence that the macromolecular conformation is a crucial factor for the fate of a polymer in the drug delivery process. Owing to the higher glomerular permeability, flexible polymers exhibit a shorter blood half‐life compared to their rigid counterparts. Additionally, more flexible polymers exhibit faster diffusion leading to their increased tumor accumulation.^34^, ^35^ The biological relevance of the chain conformation of some important biomacromolecules such as actin^36^ or DNA^37^ and the link with their functionality has been recently reported.

As a physical drug delivery tool via vascular permeabilization, FUS has emerged as a promising technique to modulate the BBB permeability due to its unique spatial targeting, noninvasiveness and deep penetration.^38^ An accumulating body of evidence^2^, ^39^, ^40^, ^41^, ^42^, ^43^ confirms the efficacy and safety of using FUS with systematically circulating microbubbles to temporarily open the BBB for enhanced drug delivery. Specifically, FUS utilizes the mechanical effects of bubble oscillations to change the vascular permeability, with the advantage that the microbubbles reduce the ultrasound intensity threshold for opening the BBB and facilitate containment of most of the permeabilization within the vasculature, thereby reducing the likelihood of irreversible neuronal damage.^38^, ^39^, ^43^ Due to the nature of vascular permeabilization, it is straightforward to relate the size of the molecules to the drug delivery outcomes. In this study, we identified another physical characteristic—the drug carrier flexibility—that could modulate the efficiency and efficacy of FUS‐enabled delivery of polymer drug conjugates across the BBB. Our findings emphasize the importance of understanding the flexibility of the drug carrier in ultrasound‐mediated drug delivery systems. Additionally, owing to the relationship of drug ratio and polymer flexibility, it is envisioned that drug ratio in the polymer conjugate may be employed as a key factor to tailor ultrasound‐based drug delivery.

Several factors may further improve the treatment outcomes. We only performed two sessions of treatments (FUS and drug administration) in our survival study. FUS‐BBB opening is currently being tested in clinical trials with multiple treatment sessions for gliomas.^44^, ^45^ It may be possible to improve the survival benefits further of an aggressive model such as GL261 with additional treatment sessions and optimized scheduling. Second, future work could further optimize the synthesis and treatment protocols based on the quantitative analyses of drug concentrations. In addition, hyaluronidase, an enzyme that breaks down HA is known to be expressed at high levels in patients with malignant glioma.^46^ Further studies are required to assess the effect of hyaluronidase in breaking down the HA carrier into smaller fragments, thereby affecting its blood circulation time, payload delivery and eventual efficacy in treating brain tumor. Lastly, delineating the cellular uptake and intracellular releasing of CPT and DOX after FUS‐enabled delivery of drug conjugates would be important for refinement of the delivery system. Future work could design this experiment by applying FUS onto a specialized BBB chip. However, ultrasound‐induced bioeffects on chips may not correlate directly with the physiological events transpiring in vivo.

## 4 CONCLUSION

Taken together, we have developed an FUS‐enhanced HA‐based platform to deliver combination chemotherapies to treat GBM. By optimizing the drug ratio in the dual‐drug‐carrying nanoconjugates, this platform was able to change the polymer flexibility, achieve effective drug delivery, and improve survival benefits in a mouse GBM model. Our results also suggest that the effectiveness of FUS‐mediated brain delivery may be dependent on the physical properties of nanocomplexes such as the flexibility, which has not been identified in previous reports.

## 5 METHODS

### 5.1 Synthesis of DOX‐HA‐CPT

Drug conjugates HA‐DOX, HA‐CPT, and DOX‐HA‐CPT were synthesized via nucleophilic acyl substitution reactions. HA of 50‐kDa MW was first dissolved at 40°C in mixture of 1:1 DI water/dimethyl sulfoxide to obtain a reaction concentration of 50 mg/ml. For activating the carboxyl groups on HA, DMAP/EDC was added to this solution at a 1:1 molar ratio (relative to the monomer mass) and was stirred for 30 min. For HA‐DOX and HA‐CPT, each drug was added dropwise at molar ratios 0.4:1 and 0.2:1 relative to the monomer mass. For the dual drug conjugates at varying ratios of DOX and CPT, each drug was added to the polymer solution based on the calculations shown in Table S1. At the end of all the reactions, the products were purified with Sephadex G‐25 PD‐10 desalting columns (5000 M.W. exclusion limit) followed by overnight dialysis (3500 MWCO) against DI water. The dialyzed product was then lyophilized and stored at 4°C, prior to reconstituting with PBS for subsequent in vitro or in vivo studies. The amounts of DOX and CPT incorporated on HA were assessed using fluorescence: Ex/Em 470/590 for DOX and 370/448 nm for CPT—for each molecule, respectively.

### 5.2 FTIR characterization

Infrared spectra were collected with NicoletTM FTIR spectrometer (Thermo Fisher Scientific, Waltham, MA) within range of 600–4000 cm^−1^ and 4 cm^−1^ spectral resolution. Lyophilized controls and DOX‐HA‐CPT conjugates were individually placed on the diamond surface of the ATR device and 32‐scan interferogram was recorded for each sample. Thermo ScientificTM OMNICTM Spectra software was used to analyze the peaks, after applying baseline, atmospheric and ATR corrections on the raw spectra.

### 5.3 Size measurements

The Malvern Panalytical NanoSight NS300 Instrument is an analytical sizing instrument. This instrument utilizes NTA to accurately characterize non‐normally size distributed particles by measuring the diffusion coefficient of each particle individually. To ensure that the instrument was sizing particles properly, 100 nm polystyrene‐latex microsphere (Catalog No. 4088, Malvern Panalytical) were suspended in PBS and pushed through the flow chamber. The laser module was then placed back into the NanoSight, and the microspheres were inspected under the recording microscope. The microscope's focus was adjusted to improve the microspheres' detection, and the instrument was then run to ensure that the reported size was within 10% deviation of 100 nm.

To analyze the conjugates, a concentrated stock of samples was diluted in ultrapure water or PBS, vortexed, and pushed into the flow chamber. The sample was then inspected under the recording microscope, adjusting the focus for optimal display of conjugates. If less than eight conjugates appeared under the microscope, the sample was deemed too dilute for accurate analysis. This procedure was repeated, with a lower dilution of the sample stock, until eight or more particles were visible under the microscope at any given time. The sample was then analyzed using the following NTA parameters: 60 s run time, and two run repetitions. Dilution factor and sample infusion rate varied among runs due to the threshold visibility of eight or more particles.

### 5.4 SAXS analysis

SAXS was used to assess the nanoparticles' architecture and dimensions. SAXS measurements were performed in a flow‐cell setup in the LiX‐16‐ID beamline at the National Synchrotron Light Source II (Brookhaven National Laboratory, Upton, NY). Scattering images were acquired each with a 1‐s exposure time. The x‐ray energy was 13 KeV. Lyophilized samples were first dissolved in DI water and then diluted by 10 times in PBS to simulate the internal salinity.

Due to low concentration of nanoparticles, we assume the structure factor S(*q*) = 1 (interparticle interactions are neglected). The scattered intensity curves were fitted using SASFit software.^47^

- *Polydisperse hard sphere model*

The following form was used to describe the spherical shell form factor,

(1) $$P\left(q\right)={\left[\frac{4}{3}\pi R^{3}\left(\text{ΔSLD}\right)3\frac{\sin\mathrm{qR}-\mathrm{qR}\cos\mathrm{qR}}{{\left(\mathrm{qR}\right)}^{3}}\right]}^{2}+\text{background}$$

where *R* is the radius of sphere and ΔSLD is the difference in scattering length densities (SLD) between particle and solvent.

Taking nanoparticles polydispersity into account, a Schulz–Zimm distribution of *R* with polydispersity parameter σ was included in the following way:

(2) $$\mathrm{SZ}=\frac{R^{Z}}{Г\left(Z+1\right)}{\left(\frac{Z+1}{<R>}\right)}^{Z+1}\exp\left[-\frac{\left(Z+1\right)R}{<R>}\right]$$

where $Z=\frac{1}{\sigma^{2}}-1$.

Since the polydispersity parameter σ and radius of sphere are correlated parameters, the σ value was set to 0.3 for all fitting procedures.

- *Generalized Gaussian coil model*
  The following form factor of Generalized Gaussian coil was used:

(3) Pggcq=I0cU12νГ12ν−Г1ν−U12νГ12νU+Г1νUνU1ν

where $U=\left(2\nu +1\right)\left(2\nu +2\right)\frac{q^{2}R_{g}^{2}}{6}$, and $Г\left(\frac{1}{2\nu }\right)$—Gamma function.

The fitting parameters for this model are *R*_g_ (gyration radius) and ν (Flory exponent).

- *Worm‐like chain model*:

The form factor of a worm‐like chain with contour length *L*, Kuhn length *A*, and diameter *d* has been described previously.^33^

### 5.5 Cell viability assay

Murine glioblastoma cells (GL261‐luc2) were kindly provided by Dr. David Reardon (Center for Neuro‐Oncology, Dana‐Farber Cancer Institute, Boston, MA) to our laboratory originally. Cells were seeded in DMEM supplemented with 10% heat inactivated FBS and 100 μg/ml G418 at 37°C in 5% CO_2_. The doubling time of GL261‐Luc2 cells ranged between 18 and 20 h and the optimal cell density used for this study (5000 cells/100 μl of media) was determined based on the expected capacity at 48 h. The 48 h incubation period ensured that drugs were left for one to two cell cycles before assessing end point viability. The next day, overnight media was replaced with fresh control media (Untreated, UT) or the treatment media with the conjugates (HA‐DOX, HA‐CPT, DOX‐HA‐CPTs) at a range of serially diluted concentrations (4.88, 19.53, 78.13, 312.5, 1250, 5000, and 20,000 nM) and left for 48 h incubation at 37°C in 5% CO_2_. Treatments for the dual‐drug conjugates were set up according to DOX concentrations. Cell Titer‐Blue® Viability Assays were performed according to the manufacturer's instructions (Invitrogen). Viability was determined as the % of live cells compared to the untreated cells. The combinational index (C.I.) was estimated based on the dose–response curves. A value of C.I. < 1 reflects synergism; C.I. equal to 1 reflects additive effect and C.I. > 1 reflects antagonism.

### 5.6 Animals and the GBM model

All animal protocols were approved by the Harvard University and Brigham and Women's Hospital Standing Committee on Animals and studies were performed in accordance with all state and federal regulations. Mice were anesthetized by intraperitoneal injections of ketamine (80 ml/kg/h) and xylazine (10 ml/kg/h) or isoflurane. Isoflurane was delivered through medical air. We used tail vein injection for i.v. administration.

To establish the GBM model, GL261‐luc2 cells (20,000 cells in 4 μl per tumor implant) were inoculated stereotactically into the Striatum (3 mm beneath the dorsal surface) of 6‐ to 8‐week‐old female C57BL/6J mice (The Jackson Laboratory). Mice were euthanized for signs of morbidity due to tumor burden.

### 5.7 FUS‐enabled BBB opening

After anesthesia, clippers and depilatory cream were used to remove the fur on the head before being placed on the FUS device. FUS (10‐ms bursts applied at 4 Hz for 100 s) was started immediately after the injection of Optison™ (GE Healthcare, Little Chalfont, Buckinghamshire, UK; dose: 100 μl/kg; diluted 4× in PBS). Based on measurements relative to the intraaural line in the previously obtained MRI, four targets in a 2 by 2 grid pattern centered on the tumor in the striatum were sonicated in each session. We started FUS immediately after the i.v. drug administration based on a previously developed drug administration protocol.^6^

A custom FUS system with cavitation‐controlled transmission was used (Figure S7A). A spherically curved, air‐backed lead zirconate titanate transducer (*f*‐number = 0.875) with a resonant frequency of 690 kHz was used. The FUS transducer was driven by a function generator (33220A, Agilent, Santa Clara, CA) and an amplifier (240 L, E&I, Rochester, NY). The acoustic beam profile was calibrated using a needle hydrophone (HNC‐0200; Onda, Sunnyvale, CA) and a house‐made radiation force balance. The width and length of the 50% isopressure contours were 3.2 and 17.4 mm, respectively. The peak negative pressure amplitude in water was 0.32 MPa without considering the skull insertion loss.

A passive cavitation detector (*f*_0_ = 1.5 MHz, 25% bandwidth) was used to record the acoustic emissions. The signal was recorded using a digitizer (3403 D, Pico Technology, Cambridgeshire, UK) after 14‐dB amplification (445 A, Stanford Research Systems, Sunnyvale, CA). All parameters were monitored and controlled in real time using in‐house developed software in MATLAB (MathWorks, Natick, MA).

### 5.8 In vivo imaging

The MRI and BLI were performed in the Brigham and Women's Hospital Research Imaging Core/Small Animal Imaging Lab (SAIL). T1‐weighted and T2‐weighted MR imaging were acquired using a 3.0‐T Bruker BioSpec® USR scanner (Bruker Corporation, Billerica, MA). BLI was acquired in the Bruker In‐Vivo Extreme II Optical/X‐ray system (Bruker Corporation, Billerica, MA), at 10 min after the i.p. injection of d‐luciferin in DPBS (150 mg/kg of body weight, Regis Technologies, Inc. Morton Grove, IL).

### 5.9 Fluorescent imaging and immunohistochemistry

Fluorescent imaging of brain sections was acquired using a fluorescence stereo zoom microscope (ZEISS Axio Zoom.V16, Carl Zeiss AG, Oberkochen, Germany). Transcardiac perfusion was performed before harvesting the brains for drug delivery assessment (Figure 3) and the histology study (Figure 5). For assessing FUS‐trigged drug delivery, mice were sacrificed 2 h after the treatment, and the harvested brains were fixed in 4% PFA for 24 h followed by 30% sucrose solution bath for cryoprotection. Brains were embedded in OCT before cryosectioning (with a thickness of 80 μm). Serial sectioning started from the dorsal surface of the brain all the way to the ventral surface. Sections were spaced with a gap of 480 μm (every six sections). A total of nine sections per brain were used for quantification and we measured the total fluorescent intensity of all the sections for each mouse.

Immunohistochemistry was performed on the paraffin‐embedded brains on the Leica Bond III automated staining platform using the Leica Biosystems Refine Detection kit. Antibody Caspase‐3 (Cell Signaling Technology, catalog # 9664, clone 5A1E) was run at 1:150 dilution with citrate antigen retrieval. Antibody CD3 (Cell Signaling technology, catalog # 99940, clone D4V8L) was run at 1:150 dilution with EDTA antigen retrieval. Antibody CD8 (Cell Signaling Technology, catalog # 98941, clone D4W2Z) was run at 1:200 dilution with citrate antigen retrieval. Antibody Ki‐67 (Biocare, catalog # CRM325, clone SP6) was run at 1:100 dilution with EDTA antigen retrieval.

### 5.10 Statistical analysis

All statistical analyses were carried out using Prism Graphpad 9.2 software. All data are presented as mean ± SEM (standard error of mean) unless specified, student's *t* test or one‐way ANOVA with Tukey's HSD analysis were used to determine significance. *p* values represent levels of significance (***p < 0.001, **p < 0.01, and *p < 0.05).

## AUTHOR CONTRIBUTIONS

**Tao Sun:** Conceptualization (equal); data curation (lead); formal analysis (lead); investigation (lead); methodology (lead); writing – original draft (lead); writing – review and editing (equal). **Vinu Krishnan:** Formal analysis (equal); methodology (equal); writing – original draft (equal). **Daniel C. Pan:** Methodology (supporting). **Sergey K. Filippov:** Formal analysis (equal); methodology (equal). **Sagi Ravid:** Methodology (supporting). **Apoorva Sarode:** Methodology (equal). **Jayoung Kim:** Data curation (supporting); methodology (supporting). **Yongzhi Zhang:** Methodology (supporting). **Chanikarn Power:** Methodology (supporting). **Sezin Aday:** Methodology (supporting). **Junling Guo:** Methodology (supporting). **Jeffrey M. Karp:** Methodology (equal); writing – review and editing (supporting). **Nathan J. McDannold:** Methodology (equal); resources (equal); writing – review and editing (equal). **Samir S. Mitragotri:** Conceptualization (equal); project administration (equal); resources (equal); writing – original draft (supporting); writing – review and editing (equal).

## CONFLICT OF INTEREST

Samir S. Mitragotri is an inventor on patents related to polymer drug conjugates (owned and managed by Harvard University). Jeffrey M. Karp has been a paid consultant and or equity holder for multiple companies (listed here https://www.karplab.net/team/jeff‐karp). The interests of Jeffrey M. Karp were reviewed and are subject to a management plan overseen by his institutions in accordance with its conflict‐of‐interest policies. Other authors declare that there are no conflicts of interest.

## Supporting information


**Appendix S1** Supporting Information

## Data Availability

The data that supports the findings of this study are available in the supplementary material of this article.
