# Supplementary material for: Ultrasound‐mediated delivery of flexibility‐tunable polymer drug conjugates for treating glioblastoma
Source: Bioeng Transl Med. 2022 Nov 19;8(2):e10408. doi: 10.1002/btm2.10408 (PMC10013755; doi:10.1002/btm2.10408)
Supplement: Supplementary file 1 — Appendix S1 Supporting Information [file BTM2-8-e10408-s001.docx]

Supplementary Materials for

Ultrasound-mediated delivery of flexibility-tunable polymer drug conjugates for treating glioblastoma

Tao Sun^1,2,3,‡^, Vinu Krishnan^1,2,‡^, Daniel C. Pan^1,2^, Sergey K. Filippov^1,†^, Sagi Ravid^3^, Apoorva Sarode^1,2^, Jayoung Kim^1,2^, Yongzhi Zhang^3^, Chanikarn Power^3^, Sezin Aday^4,5,6,7^, Junling Guo^1,2,^^, Jeffrey M. Karp^4,5,6,7^, Nathan J. McDannold^3^, Samir S. Mitragotri^1,2,*^

1. **HA-drug conjugates synthesis**

| Ratio (R) | HA (mmoles) | | DOX (mmoles) | | CPT (mmoles) | |
| --- | --- | --- | --- | --- | --- | --- |
| R2 | | 0.05 | | 0.012 | | 0.021 |
| R5 | | 0.05 | | 0.024 | | 0.021 |
| R15 | | 0.05 | | 0.024 | | 0.010 |

**Table S1.** DOX and CPT were incorporated onto HA in a series of molar ratios by varying their amounts added in the reaction mixture.

| **Ratio (R)** | **DOX (µg/ml)** | **DOX loading (wt%)** | **CPT (µg/ml)** | **CPT loading (wt%)** |
| --- | --- | --- | --- | --- |
| **R2** | 410 ± 7.65 | 5.7 ± 0.10 | 117 ± 8.7 | 1.6 ± 0.04 |
| **R5** | 736 ± 5.9 | 5.1 ± 0.04 | 94.4 ± 0.98 | 1.3 ± 0.01 |
| **R15** | 710.4 ± 12.0 | 4.9 ± 0.08 | 30.42 ± 1.5 | 0.8 ± 0.08 |

**Table S2.** Loading efficiencies of CPT and DOX.

1. **Atomic Force Microscopy (AFM) characterization**

- Sample preparation:

The drug conjugates were diluted to 2 μg/mL concentration using MilliQ water. The solutions were stirred for 1 hour at room temperature, with intermittent vortexing to ensure complete dissolution. 4 μL of the well-mixed solution was dropped on a freshly cleaved mica surface and allowed to dry over 15 minutes at room temperature, prior to imaging. This procedure was used for all samples.

- Atomic Force Microscopy (AFM) measurements:

Structure of the HA conjugates was studied using atomic force microscopy with Cypher microscope (Asylum Research, Santa Barbara, CA), in tapping mode at ambient conditions. Silicon cantilevers with chromium/gold coating (AC240TSA-R3: resonance frequencies between 44-95 kHz, and spring constant in the range of 0.3 - 4.8 N/m and 9 ± 2 mm uncoated silicon tip) from Asylum Research, Santa Barbara, CA were used for imaging in air. A scan rate between 0.7-1.0 Hz was selected, based on the frame size. Image processing and analysis was performed using Gwyddion 2.47 software (*n* = 3).


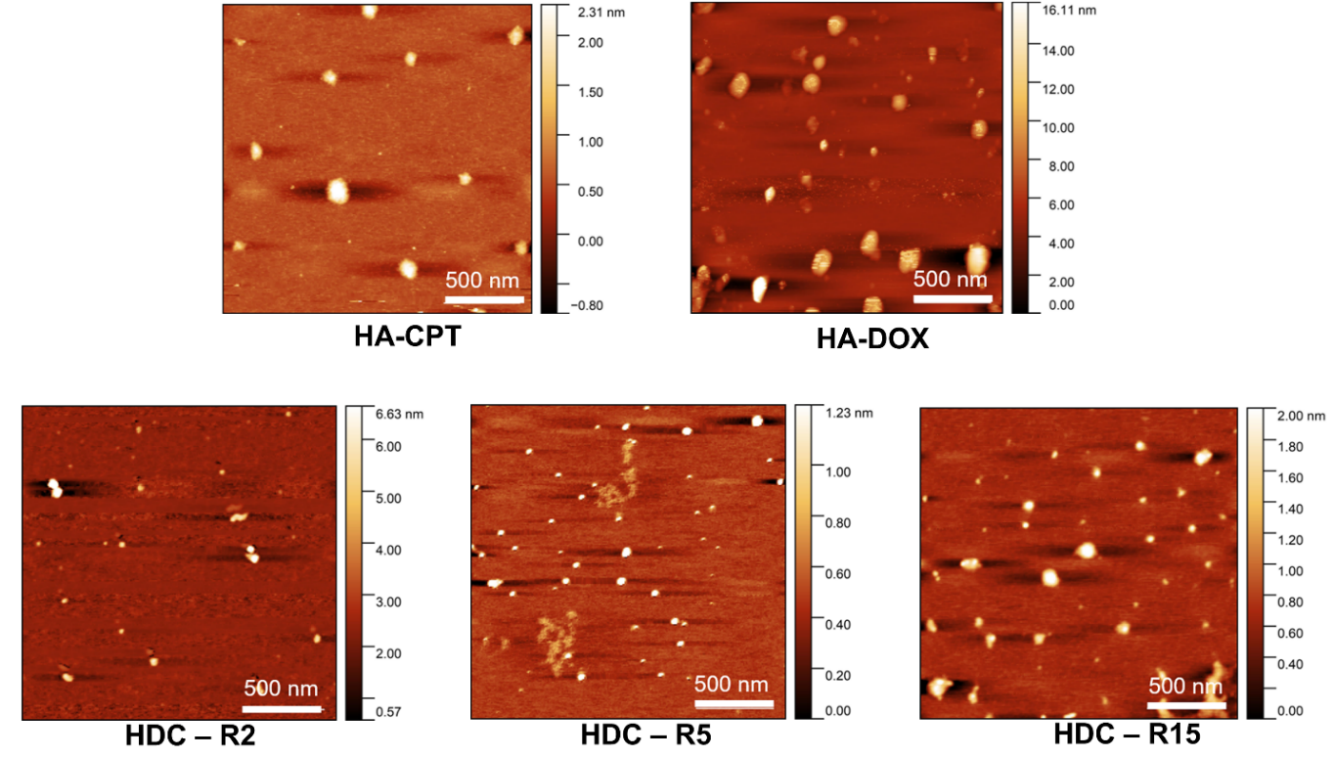


**Figure S1.** Topographic AFM images.

|  | **HA-CPT** | **HA-DOX** | **R2** | **R5** | **R15** |
| --- | --- | --- | --- | --- | --- |
| Diameter (nm) | 94.2 | 125.7 | 51.4 | 40.5 | 72.3 |
| Std. Dev. (Diameter, nm) | 43.8 | 43.8 | 14.7 | 19.0 | 36.8 |
| Height (nm) | 1.84 | 10.21 | 2.63 | 1.5 | 1.5 |

**Table S3.** Size measurements based on AFM.

1. **Nanoparticle Tracking Analyses (NTA) characterization (continued)**


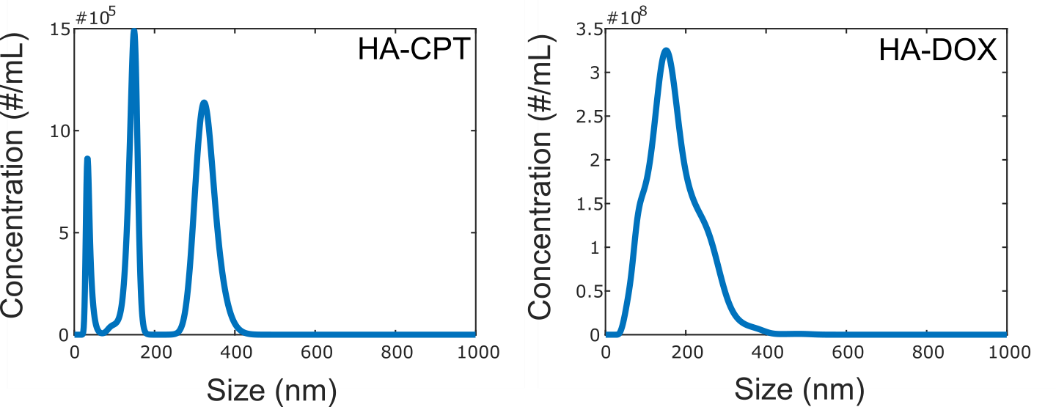


**Figure S2.** Size distribution characterization in NTA of HA-CPT and HA-DOX.

1. **Zeta-potential**

|  | **Zeta-potential (mV)** |
| --- | --- |
| **HA-CPT** | -5.020 ± 2.150 |
| **HA-DOX** | -1.520 ± 0.703 |
| **R2** | -4.340 ± 0.400 |
| **R5** | 3.600 ± 0.700 |
| **R15** | -3.050 ± 0.741 |

**Table S4.** Zeta-potentials of drug conjugates used in this work.

1. **Plasma profiles of R15**


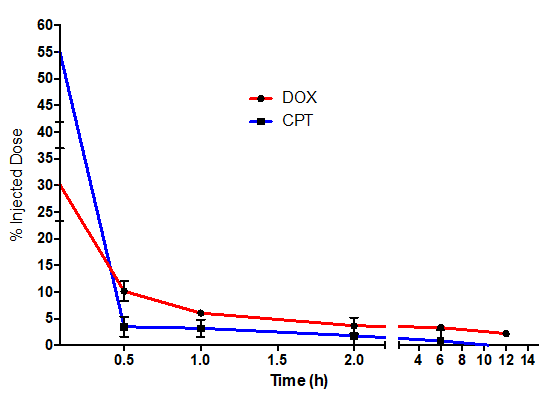


**Figure S3.** Plasma profiles of R15.

1. **Drug release profiles**

**Figure S4.** *In vitro* release rates for R2 in PBS at 37ºC. Each value represents the mean ± SEM (*n* = 3). Please refer to Fig. S2e in our recently published paper (Krishnan et al., Sci. Adv. 7, eabe6627, 2021) for the drug release profiles of R15.

1. **Biodistributions**


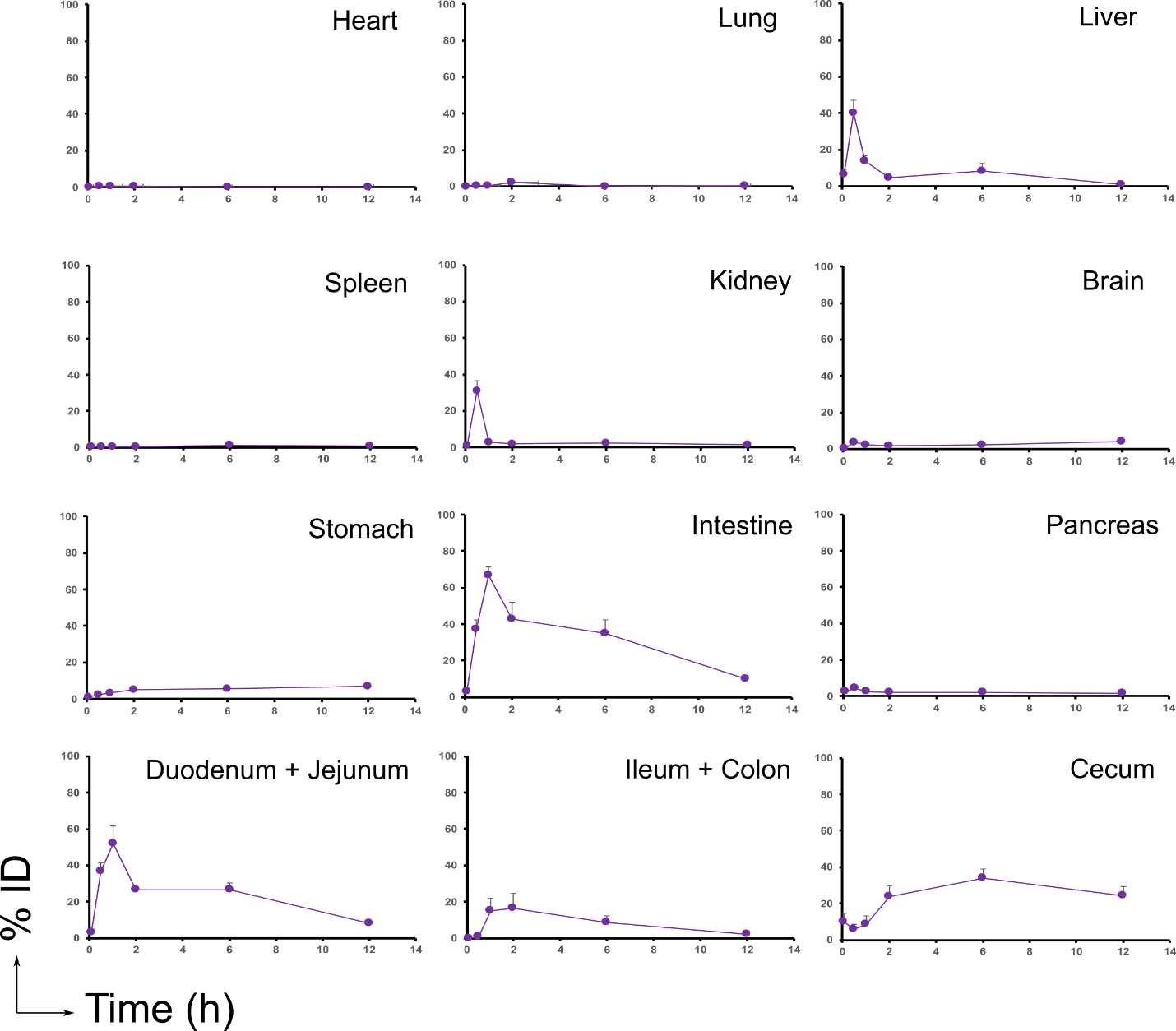


**Figure S5.** Organ biodistribution profiles of CPT for R15 *in vivo*. Each value represents the mean ± SEM (*n* = 5). ID = injected dose.


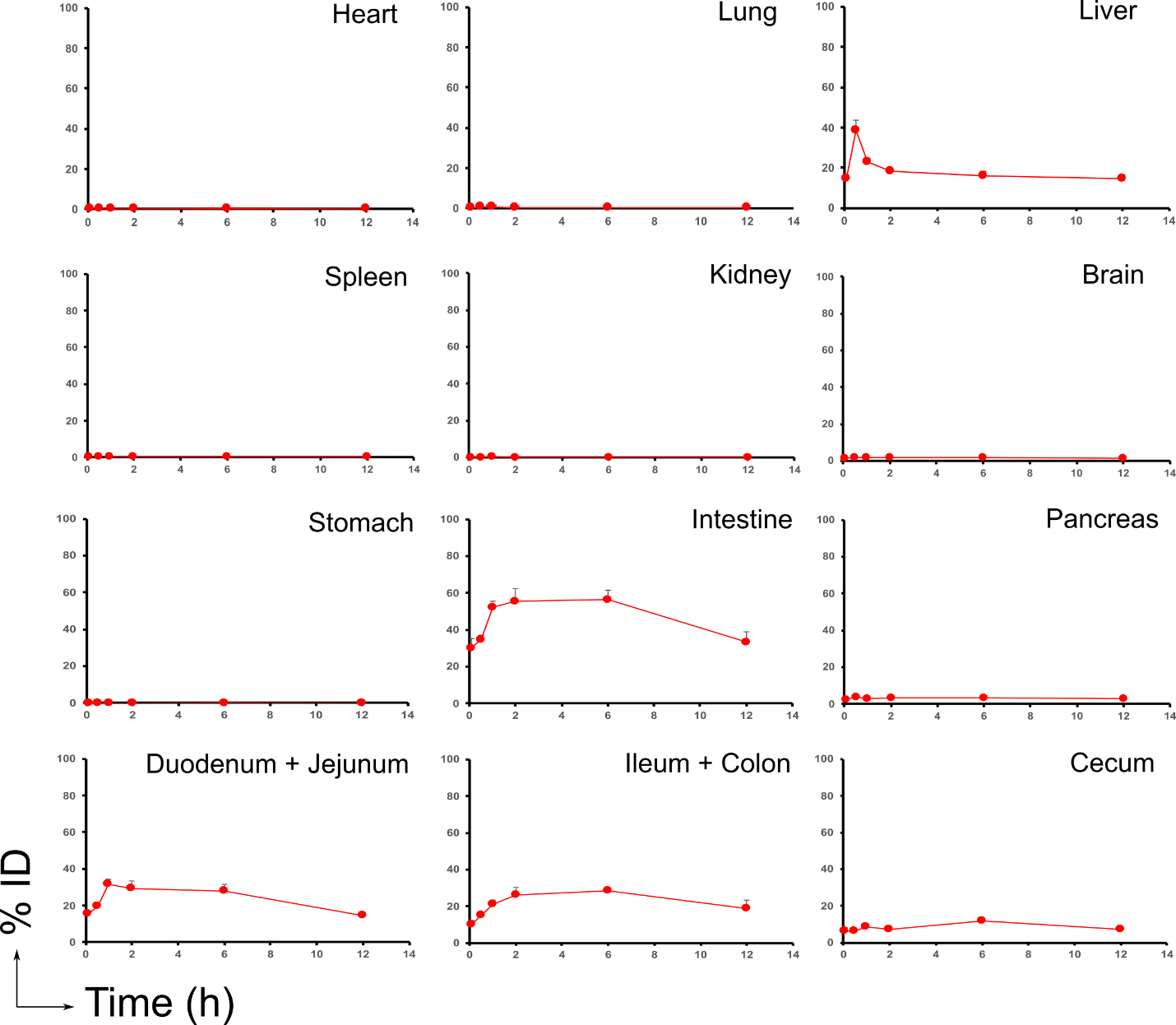


**Figure S6.** Organ biodistribution profiles of DOX for R15 *in vivo*. Each value represents the mean ± SEM (*n* = 5). ID = injected dose.

1. **FUS setup**


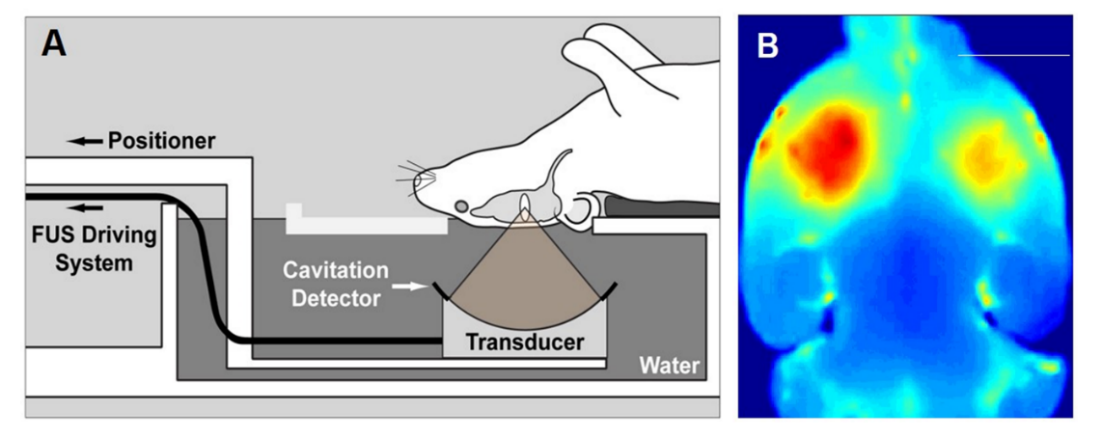


**Figure S7.** FUS-mediated BBB opening in mice. (A) The preclinical setup. (B) Mouse brain slab with bilateral tumor implantation and Trypan Blue delivery, (left) with and (right) without FUS, shows the enhanced delivery with FUS.


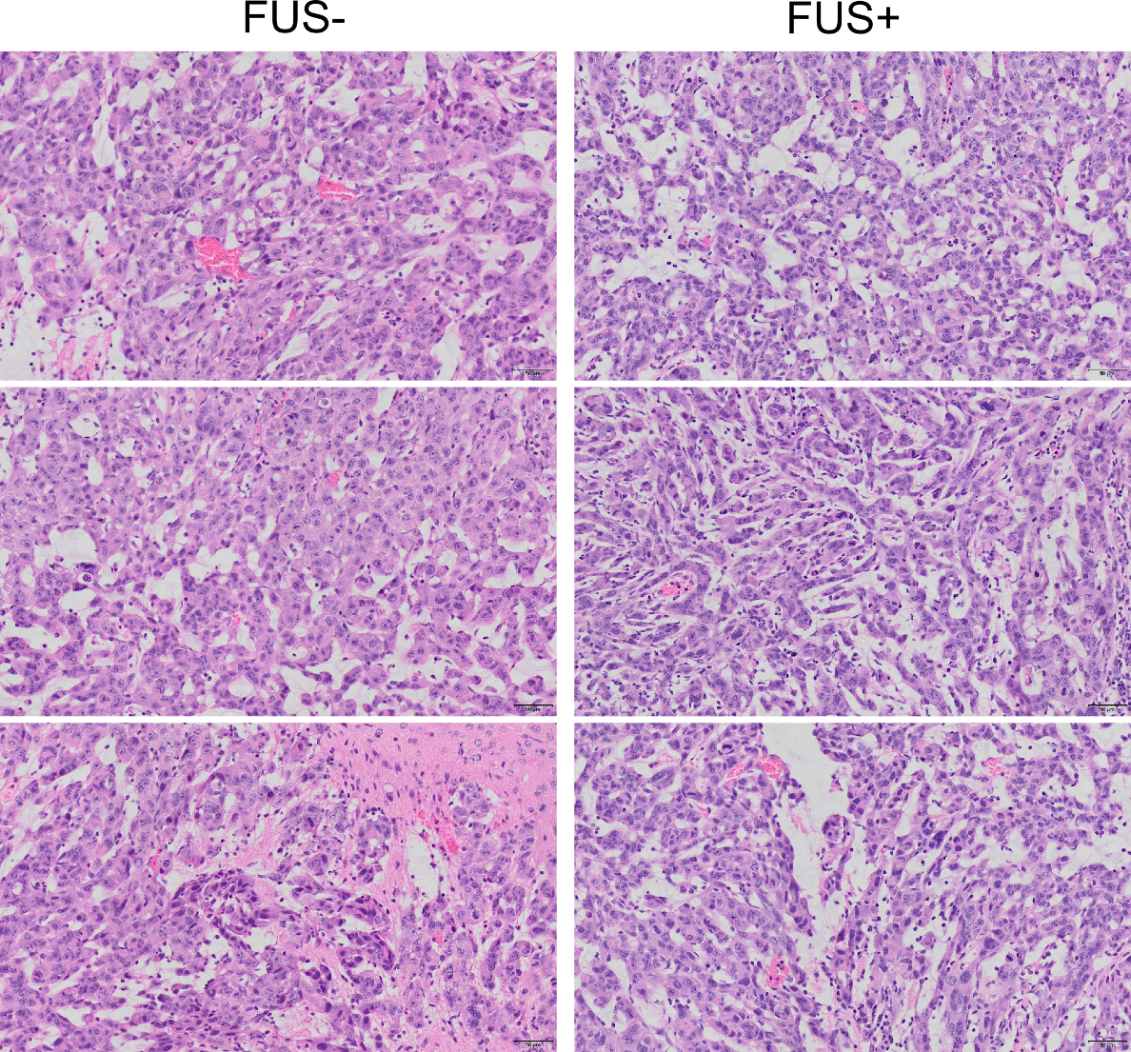


**Figure S8.** Hematoxylin and Eosin (H&E) staining using a bilateral tumor model (same animals used in Figure 5). Compared to unsonicated tumors, H&E staining results showed that FUS treatment did not induce additional extravasation of erythrocytes. Staining was performed on sections from three randomly chosen animals (out of four animals). All scale bars represent 50 µm.


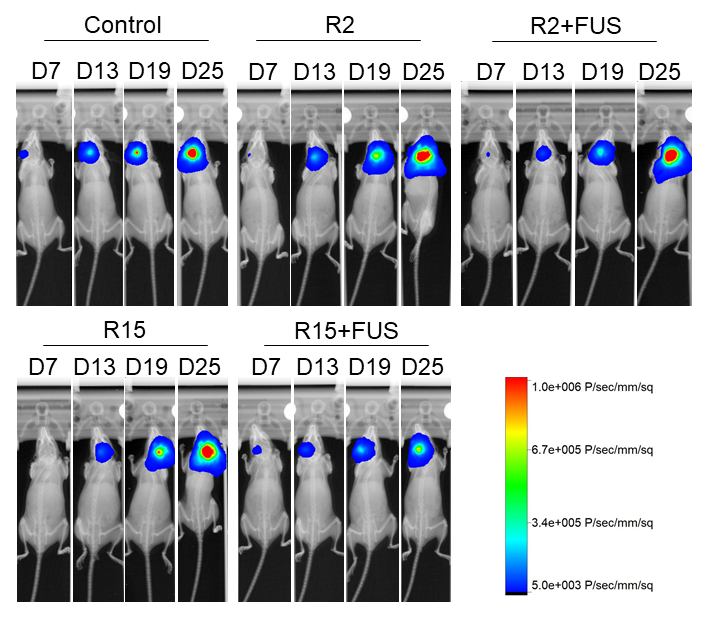


**Figure S9.** Representative bioluminescent images monitoring the tumor progression.


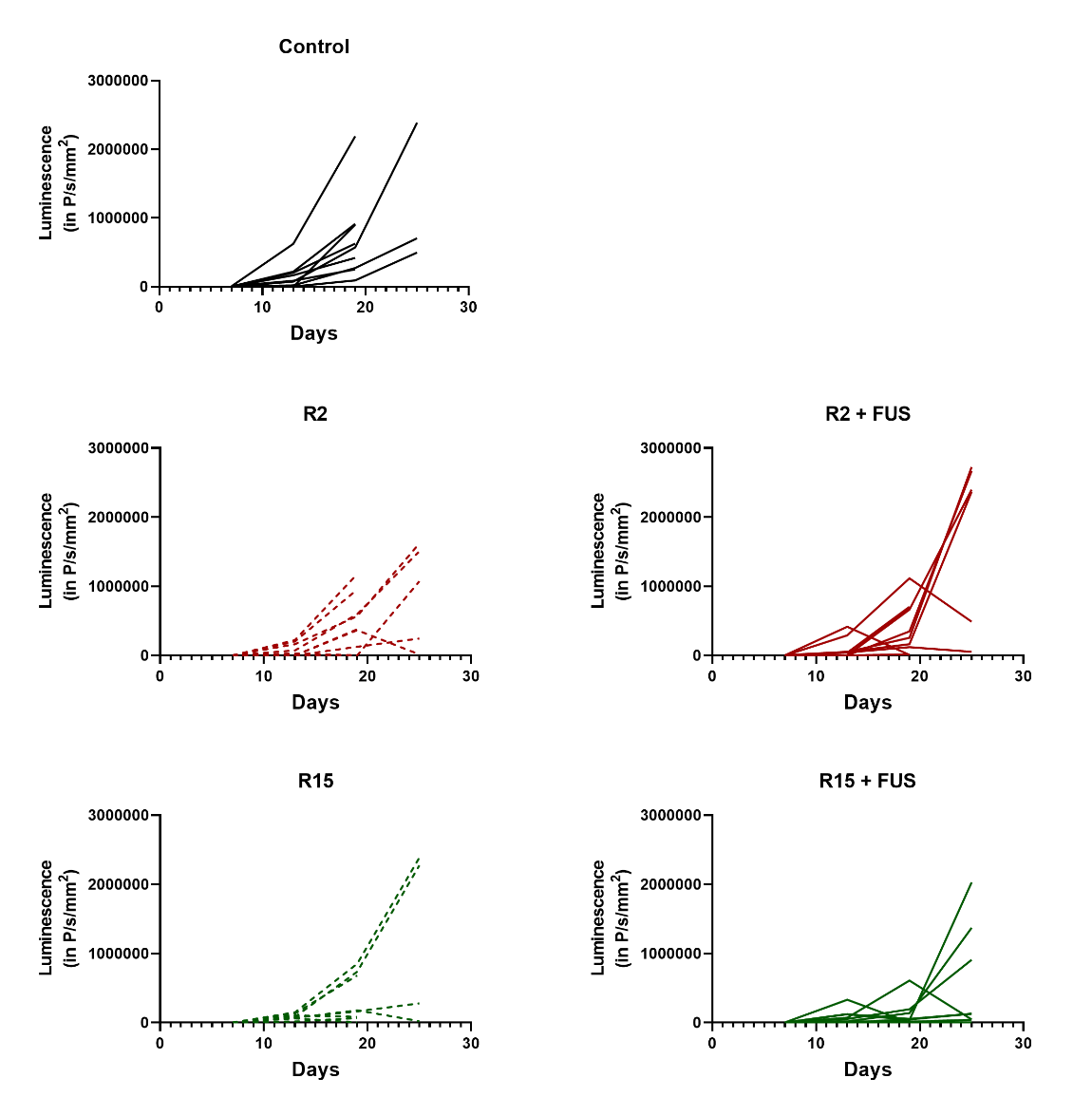


**Figure S10.** Tumor progression curves assessed by bioluminescence monitoring.
